# In Memoriam

**Published:** 1902-04

**Authors:** 


### IN MEMORIAM.

At the regular meeting of the St. Louis Dental Society, held on March 4, 1902, the following report was read and adopted.

Dr. Burt Barry was born July 26, 1871, his parents, Mr. and Mrs. L. T. Barry, of Mt. Sterling, Ill., being old settlers in Brown County, and residing there at the time of his birth. His early education was in the public schools at home and in the military schools at Salina and Orchard Lake.

In 1895 he entered the Missouri Dental College, and graduated in the class of 1898. Soon after graduating he opened an office in St. Louis. For the first year he was associated with Dr. W. W. Gardner and the remainder of the time with Dr. G. W. Loesch.

During the summer of 1901 he had a severe illness, from which he never fully recovered. Later he went to Europe; there he remained long enough to reach the belief that the London climate was best for his health, and decided to live there for at least two years. Making arrangements to that effect, he came back to this country to purchase an American dental outfit for an English dentist, with whom he expected to associate, anticipating making the move in March. On his return he spent a short time in his St. Louis office, then went to New York City, and on January 17, 1902, was married in Philadelphia to Miss Marie Peterman, a very much esteemed lady of that city. Then he went to Mt. Sterling to visit his parents and friends.

There, on the morning of February 15, he was found dead in his bed, having died without a struggle to warn his family.

His wife was in Philadelphia at the time of his death, but reached Mt. Sterling in time to attend the funeral. His remains were interred in the cemetery on the outskirts of Mt. Sterling, on February 18, 1902. His wife, parents, one brother, and three sisters survive him.

Dr. Barry, soon after graduating, became a member of our Society, and just before his severe illness was preparing a paper on Dental Materia Medica, to be read before the Society.

In college, "Burt," as the boys called him, was studious and industrious, aiming to make the most of his opportunities, keeping up his studious habits after entering practice.

Dr. Barry was prominent in the Jefferson Club during its early history. Personally he was affable, courteous, and gentlemanly always, and he made friends readily. He was fond of athletics, in several branches of which he was skilled.

John G. Harper,

F. F. Fletcher,

O. H. Manhard,

*Committee.*

